# Supplementary material for: Support Vector Regression Modelling of an Aerobic Granular Sludge in Sequential Batch Reactor
Source: Membranes (Basel). 2021 Jul 22;11(8):554. doi: 10.3390/membranes11080554 (PMC8400290; doi:10.3390/membranes11080554)
Supplement: Supplementary file 1 [file membranes-11-00554-s001.zip › membranes-1262585-supplementary.pdf]

Table 1 Normalized data set for SBR30^{o}C

Day	COD		TOC	TP	TN	AN	Biomass
	**Influent (mg/L)**	**Effluent (mg/L)**	**Influent (mg/L)**	**Influent (mg/L)**	**Influent (mg/L)**	**Influent (mg/L)**	**MLSS (g/L)**
1	0.5	0.652	0.666	0.5	0.666	0.5	0.2404
3	0.5	1	0.666	0.5	0.666	0.5	0.139
6	0.5	0.507	0.666	0.5	0.666	0.5	0.050
9	0.5	0.420	0.666	0.5	0.666	0.5	0.088
12	0.5	0.615	0.666	1	0.666	0	0
15	0.5	0.253	1	1	1	0	0.075
18	0	0.326	1	1	1	0	0.151
21	0	0.239	1	1	1	0	0.227
24	0	0.202	1	1	1	0	0.405
27	0	0.130	1	0	0.944	0	0.443
30	0	0.086	1	0	0.944	0.5	0.481
33	0	0.072	0	0	0.944	0.5	0.531
36	0	0.086	0	0	0.944	0.5	0.594
39	0	0.108	0	0	0.944	0.5	0.556
42	1	0.072	0	0.5	0.962	0.5	0.607
45	1	0.057	0.444	0.5	0.962	1	0.645
48	1	0.036	0.444	0.5	0.962	1	0.683
51	1	0.043	0.444	0.5	0.962	1	0.759
54	1	0.014	0.444	0.5	1	1	0.848
57	1	0.007	0.444	0.5	1	1	0.974
60	1	0	0.444	0.5	1	1	1
